# Association between dietary inflammatory index and body fat percentage among newly diagnosed breast cancer patients

**DOI:** 10.1080/07853890.2024.2303399

**Published:** 2024-01-19

**Authors:** Wai Han Ng, Zalina Abu Zaid, Barakatun Nisak Mohd Yusof, Syafinaz Amin Nordin, Poh Ying Lim

**Affiliations:** a Department of Dietetics, Faculty of Medicine and Health Sciences, Universiti Putra Malaysia, Serdang, Selangor, Malaysia; b Department of Dietetics and Food Service, National Cancer Institute, Ministry of Health, Putrajaya, Malaysia; c Department of Dietetics, Hospital Sultan Abdul Aziz Shah, Universiti Putra Malaysia, Selangor, Malaysia; d Department of Medical Microbiology, Faculty of Medicine and Health Sciences, Universiti Putra Malaysia, Serdang, Selangor, Malaysia; e Department of Community Health, Faculty of Medicine and Health Sciences, Universiti Putra Malaysia, Serdang, Selangor, Malaysia

**Keywords:** Dietary inflammatory index, obesity, breast cancer, body fat percentage, body composition

## Abstract

**Background:**

Obesity, particularly excessive body fat, is an established risk factor and substantial prognostic determinant in breast cancer. Recent studies suggested that diet-related inflammation plays a key role in obesity. This study aimed to determine the association between energy-adjusted dietary inflammatory index (E-DII) and body composition, particularly body fat percentage, among patients with newly diagnosed breast cancer.

**Materials and methods:**

This cross-sectional study was conducted on 124 breast cancer outpatients within the first year of diagnosis and yet to commence oncological treatment. Body composition parameters [body weight, body mass index (BMI), body fat percentage, fat mass over fat-free mass ratio (FM/FFM), muscle mass, and visceral fat] were obtained using a bioelectrical impedance analyzer. Body fat percentage was categorized into two groups which were normal (<35%) and high (≥35%). The E-DII was calculated from the validated 165-items Food Frequency Questionnaire (FFQ) and categorized into three groups or tertiles. Multiple logistic regression analysis was used to determine the association between the E-DII and body fat percentage.

**Results:**

Mean body weight, body fat percentage, FM/FFM, and visceral fat increased as E-DII increased from the lowest tertile (T1) to the most pro-inflammatory tertile (T3) (p for trend <0.05). E-DII was positively associated with body fat percentage (OR 2.952; 95% CI 1.154–7.556; *p* = 0.024) and remained significant after adjustment for cancer stage, age, physical activity, ethnicity, smoking history, and presence of comorbidities. Compared to T1, participants in T3 had a significantly lower consumption of fiber, vitamin A, beta-carotene, vitamin C, iron, thiamine, riboflavin, niacin, vitamin B6, folic acid, zinc, magnesium, and selenium, but a higher intake of total fat, saturated fat, and monounsaturated fatty acids.

**Conclusions:**

A higher E-DII was associated with increased body fat percentage, suggesting the potential of advocating anti-inflammatory diet to combat obesity among newly diagnosed breast cancer patients.

## KEY MESSAGES

E-DII was significantly and positively associated with high body fat percentage, particularly among pre-menopausal breast cancer patients.

Participants in the highest E-DII tertile group had significantly higher body weight, body fat percentage, FM/FFM, and visceral fat than those in the lowest E-DII tertile group.

Those in the highest E-DII tertile group were more likely to consume a higher intake of total fat, saturated fat, and monounsaturated fatty acids, but lower fiber, vitamin A, beta-carotene, vitamin C, iron, thiamine, riboflavin, niacin, vitamin B6, folic acid, zinc, magnesium, and selenium compared to the lowest E-DII tertile group.

## 1. Introduction

As the most commonly diagnosed cancer among female globally, breast cancer is a significant health challenge worldwide with 2.3 million newly diagnosed cases and 685,000 deaths in year 2020 alone [1]. Similarly, obesity is a rising health crisis with an estimated of one billion people worldwide, including one in every five women to be living with obesity by 2030 [2]. Many previous studies have tried to comprehend the relationship between obesity and breast cancer, with the first study back in 1976 reported that, compared to their normal weight counterparts, obese breast cancer patients with body weight >20% over their standard weight calculated by 0.9 × (height in cm − 100) had larger tumours, greater lymphatic invasion rates, and poorer overall survival [3]. Today, obesity particularly excessive body fat, is an established risk factor for breast cancer diagnosis [4] and a substantial determinant of prognosis, correlating to a 35% to 40% increased risk of recurrence and mortality regardless of menopausal or hormone receptor status [5].

Emerging evidence suggests that chronic inflammation is instrumental in undergirding the association between obesity and breast cancer. Inflammation is a process by which the immune system protects against foreign bodies and aids in the repair of damaged tissue [6]. Nevertheless, if it is not regulated properly but remains prolonged, it would be known as chronic inflammation leading to the development of diseases, including breast cancer [7]. Low-grade chronic inflammation is a hallmark of obesity [8]. Excess adipose tissue fosters a chronic systemic inflammatory microenvironment by producing a multitude of cytokines and acute-phase proteins such as interleukin-6 and C-reactive protein, which activate pro-inflammatory signalling pathways and prompt tumour development and progression [9]. Obesity is also linked to white adipose tissue breast inflammation capable of activating the nuclear factor kappa B pathway and the production of oestrogens by the enzyme aromatase, contributing to tumour development and progression [10,11]. Obese women and those who gain weight within the first to third years of breast cancer diagnosis have a higher chance of recurrence than lean women or those who do not gain weight [12].

Obesity is an abnormal and excessive accumulation of fat in the body due to overeating and under-exercising [13,14]. Various dietary factors in obesity have been studied in the past, such as excessive carbohydrate intake, high-fat consumption, and inadequate fibre intake [15]. Recent studies also suggest that diet-related inflammation may play a key role in obesity [16]. As a typical human diet consists of a variety of both pro-inflammatory and anti-inflammatory foods and nutrients, the dietary inflammatory index (DII) has been developed and validated as a nutritional tool to study the inflammatory capacity of the diet as a whole rather than single nutrient [17,18]. Higher DII scores indicate a more pro-inflammatory diet, whereas lower DII scores indicate a more anti-inflammatory diet [17]. DII is directly related to adipose tissue, particularly abdominal obesity in women, and is inversely associated with fat-free mass [19,20]. As obesity can be a consequence of a pro-inflammatory diet and simultaneously exert inflammatory effects on cancer progression, a bidirectional association between inflammation and obesity has been proposed [21].

Therefore, this study aimed to determine the association between DII and body composition, particularly body fat percentage, in a newly diagnosed breast cancer population who had yet to commence any oncological treatment in Malaysia. We hypothesized that a higher DII score is associated with an increased body fat percentage. Determining the role of an inflammatory diet in obesity could serve as the basis for future interventional studies intended to improve body composition and address obesity problems among patients with breast cancer.

## 2. Materials and methods

### 2.1. Study design and participants recruitment

**Figure 1.**
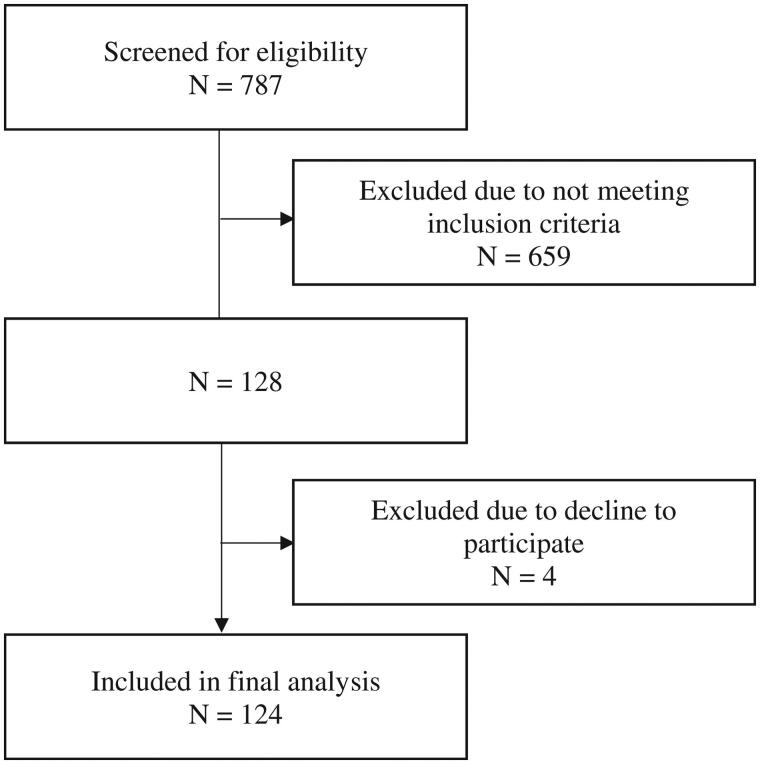
Flow chart of study participants.

A total of 124 female pre-treatment breast cancer patients were recruited during outpatient visits using universal sampling in this cross-sectional study conducted at the National Cancer Institute (NCI), Putrajaya, Malaysia from May to November 2022 (Figure 1). The NCI is the national referral centre for oncology patients, catering to all new and follow-up oncology cases referred from both government and private health institutions all over Malaysia. Ambulatory Malaysian females who were aged over 18 years old, within the first year of breast cancer diagnosis confirmed with biopsy and yet to start on any oncological treatment be it chemotherapy, radiotherapy, hormonal or immunotherapy were included in the study. Those who were pregnant or on lactation, had medical conditions requiring fluid restriction or severe dietary restriction such as renal disease, complicated with fluid retention illness, or having symptoms of oedema and ascites that could affect body composition analysis accuracy, were excluded from the study. None of the participants reported any implausible total energy intake (<600 kcal/day or >3500 kcal/day). This study was registered in the National Medical Research Registry (NMRR) Malaysia, and ethical approval was received from the Medical Research and Ethics Committee (MREC) of the Ministry of Health, Malaysia [NMRR ID-22-00219-FBX (IIR)] followed by site approval from National Cancer Institute, Putrajaya, Malaysia [IKN.CRC/760-2/4/1 JLD.2 (55)]. Verbal and written informed consent were obtained from all participants prior to the assessment.

### 2.2. Sample size calculation

The sample size was estimated using two population mean formula [22]. Prior data indicated that the mean body fat percentage of the lower energy-adjusted DII (E-DII) group was 32.7 (standard deviation = 5.0) and the mean body fat percentage of the higher E-DII group was 36.0 (standard deviation = 5.1) [23]. Thus, a minimum sample size of 100 was able to reject the null hypothesis with a probability (power) of 0.90. The Type I error probability associated with this test of null hypothesis was 0.05. With an additional 20% dropout rate, the required sample size was 120.

### 2.3. Anthropometric measurement and body composition data

All anthropometric measurements were performed by the same trained dietitian using standardized protocols prior to the interview session. A stadiometer (Seca 222, Medical Scales & Measuring Systems Seca, United Kingdom) was used to measure the body height to the nearest of 0.1 cm. Calibrated bioelectrical impedance analysis (BIA) scale, which was the Total Body Composition Analyzer (TANITA) model SC-330MA (Tanita Corporation, Tokyo, Japan) with an accuracy of up to 0.1% for body fat percentage, and up to 0.1 kg for body weight, fat mass, fat-free mass, muscle mass, and visceral fat was used to measure body weight and body composition components. All measurements were taken with the participants being shoeless and wearing lightweight clothing with empty pockets, without watches or other accessories, standing upright with front facing on the metal plate of the scale. Body mass index (BMI) was calculated as the actual body weight (kg) divided by the square of height (m^2^) [24]. Body fat percentage was categorized as normal (<35%) or high (≥35%) [25]. Fat mass over fat-free mass ratio (FM/FFM) was calculated as fat mass (kg) divided by fat-free mass (kg).

### 2.4. Dietary assessment and dietary inflammatory index (DII)

Dietary assessment of the participants was performed by a trained dietitian *via* face-to-face interviews using the Food Frequency Questionnaire (FFQ) adapted from the Malaysian Adults Nutrition Survey, National Health and Morbidity Survey (NHMS), 2014 [26]. The FFQ consisted of 165 food items that were listed in 15 food groups, including cereal products, meats, fish and seafood, eggs, nuts and legumes, milk and dairy products, fast food, vegetables, fruits, beverages, confectionaries, condiments, bread spread, cooking oil, and alcoholic beverages. The participants were requested to provide answers on (1) the type of normally consumed food items, (2) the number of serving sizes consumed based on the standard serving size, and (3) the frequency of consumption for each food item in the year prior to the interview by choosing only one option out of the four options provided in the FFQ, which were number of times per day, number of times per week, number of times per month, and never. Each food listed was given a standard serving size based on the Atlas of Food Exchanges and Portion Sizes [27], using food models and common household measures such as teaspoons, tablespoons, bowls, cups, and matchbox sizes. The nutrient intakes of the participants were analysed using Nutritionist Pro™ Diet Analysis Software version 3.1.0 (Axxya Systems, 2023). Energy and nutrient data were obtained from the Nutrient Composition of Malaysian Foods database in the Nutritionist Pro software. If a food or beverage was not listed in the Malaysian food database, data from the Singapore Food Composition database and the U.S. Department of Agriculture database (USDA) were included. Nutrient information from branded food products in Malaysia was also included if it was unavailable in all databases.

FFQ-derived nutrient analysis was then used to calculate the DII scores for each participant. DII was developed to quantify the inflammatory potential of individuals’ diets on a scale from maximally anti-inflammatory (most negative score) to maximally pro-inflammatory (most positive score). A detailed description of the DII development and validation is described elsewhere [17]. In this study, 24 of the 45 food parameters were used, including pro-inflammatory parameters (energy, protein, carbohydrate, total fat, saturated fat, cholesterol, iron, and vitamin B12) and anti-inflammatory parameters (monounsaturated fatty acids [MUFA], polyunsaturated fatty acids [PUFA], vitamin A, beta carotene, vitamin C, thiamine, riboflavin, niacin, vitamin B6, folic acid, zinc, magnesium, selenium, fibre, caffeine, and alcohol). Some food components were not included in the present study because they were not used in the Malaysian diet, such as ethanol, or data were not available, such as some spices. Energy-adjusted DII (E-DII) scores were calculated using the density approach by calculating the DII per 1000 kcal consumption. For E-DII, energy was in the denominator; therefore, 23 parameters were used for the computation. The analysed nutrient data were translated into z-scores using an energy-adjusted global comparison database [28,29] by subtracting the mean of the global database from the individual’s self-reported value and then dividing by the standard deviation. These scores were then converted to proportions (with values ranging from ‘0’ to ‘1’) and centred on zero by doubling each and subtracting ‘1’. These centred proportions were then multiplied by their respective coefficients (overall food parameter-specific inflammatory effect scores) and summed to obtain the overall E-DII score. For analytical purposes in this study, E-DII scores were categorized into three groups (tertiles), with the lowest and highest E-DII scores found in the first and third tertiles, respectively.

### 2.5. Other study variables

#### 2.5.1. Socio-demographic and medical characteristics

Sociodemographic information including age, ethnicity, education level, household income level, and marital status, as well as medical characteristics including menopausal status, smoking history, cancer staging, and comorbidities were obtained from the computerized Hospital Information System and through face-to-face interviews using a standardized data collection form. Comorbidities were defined based on the official record of a formal diagnosis by a doctor in the Hospital Information System.

#### 2.5.2. Biochemical parameters

All biochemical parameters including serum albumin, haemoglobin, white blood cells, neutrophil and lymphocyte counts, were obtained through venous blood sampling following the standardized procedures by registered nurses in NCI and analysed in the NCI Pathology Department.

#### 2.5.3. Physical activity level

Physical activity levels were assessed using the short-form International Physical Activity Questionnaire which provides information on the frequency, duration, and intensity of physical activity [30]. Vigorous physical activity, moderate physical activity, walking, and sitting in the past 7 days were recorded using this questionnaire. Metabolic Equivalents (METs) were then calculated to estimate the total physical activity per week for each participant. Using the instrument’s scoring protocol, physical activity was categorized into three groups: sedentary, moderate, and high, based on a combination of frequency of activity, duration of each activity, and MET minutes per week for all activity types.

#### 2.5.4. Handgrip strength

Handgrip strength was measured using a Jamar hand dynamometer (Fred Sammons Inc., Burr Ridge, Illinois, USA). Participants were seated comfortably in an upright position with the shoulder adducted and neutrally rotated, elbow flexed at 90°, forearm and wrist in a neutral position. They were asked to hold the dynamometer and were instructed to squeeze the dynamometer with maximum force and maintained for five seconds. The procedure was repeated three times with the dominant hand and recorded in kilograms. The average reading of three consecutive attempts was used as the final handgrip strength result.

### 2.6. Statistical analysis

All analyses were performed using the statistical software IBM SPSS (version 27.0; SPSS Inc., Chicago, IL, USA). The Kolmogorov–Smirnov test was used to test the normality of all continuous variables. Participants were grouped based on tertiles of the E-DII. To compare the general characteristics among the E-DII tertiles, Kruskal–Wallis and chi-square tests were used for continuous and categorical variables, respectively. The Jonckheere–Terpstra test was used to evaluate trends in p-values for continuous data. Analysis of variance (ANOVA) was used to test for mean differences in the distribution of dietary intake and specific food groups across the E-DII tertiles. Odds ratios (OR) and 95% confidence intervals (CI) were estimated for body fat percentage as the outcome variable and the E-DII as the independent variable using logistic regression models. No covariates were adjusted for in model 1. For model 2, cancer stage was adjusted. In model 3, further adjustments for age, physical activity, ethnicity, smoking history, and comorbidities including no known medical illness (NKMI), type 2 diabetes mellitus (T2DM), hypertension, and dyslipidemia were performed. Further analysis was done by stratifying participants into pre- and post-menopausal groups and the same logistic regression models was repeated for both groups. The level of statistical significance was set at *p* < 0.05.

## 3. Results

### 3.1. Description of sociodemographic and medical characteristics of study participants

The sociodemographic and medical characteristics of the 124 study participants based on the E-DII tertile grouping are shown in Table 1. Overall, the E-DII scores in this study ranged from −2.85 (most anti-inflammatory) to +3.53 (most pro-inflammatory). The overall median age of the participants was 50 years (range, 27–72 years). No significant differences were detected in age, education level, ethnicity, marital status, household income, smoking history, or menopausal status among the E-DII tertiles. However, it is worth mentioning that, in terms of comorbidity, the number of participants with diabetes increased across the E-DII tertiles from T1 (most anti-inflammatory) to T3 (most pro-inflammatory). A similar pattern was observed for hypertension, where 40.5% of participants with hypertension were in T3, compared to only 26.2% in T1. At the same time, T1 consisted of most participants without any comorbidity (38.0%) compared to T3 (26.8%). There were more participants with stage 1–2 breast cancer in T1, whereas T2 and T3 consisted of more participants with a stage 3–4 diagnosis.

### 3.2. Body composition, biochemical profile, physical activity and functional status of participants

All body composition parameters showed an increasing trend as E-DII increased from T1 to T3 (Table 2). Study participants were generally in the overweight category (BMI 25.0–29.9 kg/m^2^) with elevated body fat percentage (≥35.0%). However, the participants’ mean body weight, body fat percentage, muscle mass, and FM/FFM increased significantly as E-DII scores increased from T1 to T3 (*p* < 0.05). The same pattern was observed for visceral fat, which increased as the E-DII score increased (*p* < 0.05). As for physical activity level, 42.9% of those with sedentary activity levels were in T1, while 37.1% of those with moderate-to-high activity levels were in T3, but there was no significant difference among the E-DII tertiles. Functionally, the study participants had low handgrip strength (<18 kg), but no significant trend was observed. Regardless of E-DII tertiles, participants were generally well nourished with albumin median score of 40.0 to 41.0 g/dL and hemoglobin median score of 12.5 to 12.7 g/dL. Nevertheless, there was a significant increase in the white blood cell (WBC) count from T1 to T3 (*p* < 0.05) and lymphocytes (*p* < 0.01).

### 3.3. Dietary inflammatory index score with dietary intake among participants

The distribution of the 23 food parameters across E-DII tertiles is shown in Table 3. In terms of macronutrients, there was a significant difference in fat intake, with the highest fat intake at T3 and the lowest fat intake at T1 (*p* < 0.05). Compared to participants in T1, those in T3 had a significantly higher intake of saturated fat (SFA) (*p* < 0.01) and monounsaturated fatty acid (MUFA) (*p* < 0.05). No significant differences were observed in carbohydrate and protein intake. Higher E-DII scores were also significantly related to lower intakes of vitamin A, beta-carotene, vitamin C, thiamine, riboflavin, niacin, folic acid, zinc, magnesium, and fibre (*p* < 0.01). No significant differences were observed in polyunsaturated fatty acid (PUFA), cholesterol, vitamin B12, or alcohol intake across the E-DII tertiles.

### 3.4. Association between E-DII tertiles and body fat percentage

The association between the E-DII tertiles and body fat percentage is shown in Table 4. The highest E-DII tertile was positively associated with body fat percentage (OR 2.952; 95% CI 1.154–7.556; *p* = 0.024) and remained significant after adjustment for cancer stage, age, physical activity, ethnicity, smoking history, and presence of comorbidities, including type 2 diabetes mellitus, hypertension, and dyslipidaemia (OR 3.996; 95% CI 1.311–12.174; *p* = 0.015). However, when stratified according to menopausal status, it was found that the association between E-DII tertiles and body fat percentage was only significant for pre-menopausal (OR 4.583; 95% CI 1.038-20.240; *p* = 0.045) but not post-menopausal participants as shown in Table 5.

## 4. Discussion

The main finding of this study was that breast cancer patients in the highest E-DII tertile group had less favourable body composition linked with obesity, with significantly higher body fat percentage, FM/FFM ratio, and visceral fat compared to those in the lowest E-DII tertile group. Although the BMI differences were not statistically significant, an increasing trend was observed across the E-DII tertiles. Furthermore, BMI is unable to distinguish between fat mass and lean body mass, rendering it a controversial tool with limited diagnostic performance to determine obesity [31], especially in the overweight group with a BMI between 25 and 30 kg/m^2^ [32], which was the majority of our study population. In comparison, body fat percentage has been suggested to have breast cancer prognostic value [33], in which higher adiposity is associated with all-cause mortality, breast cancer-specific mortality, recurrence, and distant recurrence in breast cancer patients, regardless of menopausal status [34]. A previous study among breast cancer survivors also found that those who were obese with body fat ≥35% had significantly higher levels of inflammatory markers of C-reactive protein and serum amyloid A protein than their counterparts with body fat <35% [35]. Therefore, among all the studied body composition variables, body fat percentage was chosen as the obesity endpoint when determining its association with DII.

In our study, there was a significant increase in most obesity indices across the E-DII tertile, from most anti-inflammatory to most pro-inflammatory. The highest E-DII tertile, which denotes a pro-inflammatory diet, remains an independent risk factor for obesity indicated by body fat ≥35%, even after adjustment for covariates of cancer stage, age, physical activity, ethnicity, smoking history, and presence of comorbidities including NKMI, T2DM, hypertension, and dyslipidemia. This finding is consistent with that of most previous studies. A six-month randomized controlled trial among 81 obese adults found that there was a significant reduction in body weight and visceral fat among participants subjected to an anti-inflammatory diet compared to the control group with an isocaloric diet [36]. In the SUN cohort study with a median follow-up of 8.1 years, the odds ratio for overweight and obesity was 1.32 (95% CI 1.08-1.60) for those in the highest DII quartile compared to the lowest quartile [37]. In the same study, a pro-inflammatory diet was significantly correlated with yearly weight gain [37]. A similar finding was observed in another study where higher DII scores increased the odds of obesity or overweight by 2.5 times even after adjusting for major confounding variables [38]. Nonetheless, most of the previous studies focused on the pre-morbid obese adult population, but the current study is one of the rare studies that focus on pre-treatment breast cancer women in view of the prominent impact of obesity and excessive body fat in this population.

However, when participants were stratified according to menopausal status, it was found that the association between E-DII tertiles and body fat percentage was only significant for pre-menopausal breast cancer patients, but not post-menopausal participants. This is in contrast to a previous study namely PREDIMED trial where a significant positive correlation was observed between the DII score and BMI as well as waist circumference among 4145 women aged 60-80years old [20]. Besides, a cohort study on 34,700 postmenopausal women over a period of 25 years follow-up found positive associations between DII scores and breast cancer risk with stronger associations in obese women [39]. A study among 1,344 postmenopausal Korean women also found that higher DII scores were associated with increased odds of the osteosarcopenic obesity (OR = 2.186, 95% CI: 1.182–4.044, *p* < 0.05) [40]. These differences in analysis outcome might be due to small sample size of post-menopausal group in the current study compared to the large-scale sample of previous studies.

Growing evidence suggests that there is a mutual bidirectional association between body fat and inflammation [41]. Although adiposity promotes the production of inflammatory cytokines, a pro-inflammatory diet can sustain and increase body fat, thereby producing a vicious cycle [9,20]. Our study supports this theory as significantly higher white blood cell and lymphocyte counts across the E-DII tertiles might reflect the ongoing subtle chronic inflammation effect from such a pro-inflammatory diet although the exact mechanism between DII and body fat remains uncertain, it was found that a more pro-inflammatory diet was associated with higher leptin and a lower adiponectin to leptin ratio [42], and there is a direct association between leptin levels and fat mass in obese individuals. Leptin also binds to the long isoform receptor expressed by various immune cell types and induces inflammatory responses that promote chronic inflammation [43]. However, adiponectin, which suppresses inflammation, is drastically reduced in obese individuals [44]. Recent studies have also examined the role of the gut microbiome and found that obese individuals have a less diverse gut microbiota [43]. The consumption of dietary fiber (an anti-inflammatory food parameter in DII) that cannot be fully digested by human enzymes is catabolized by the gut microbiota, producing short-chain fatty acids that have a beneficial role in central appetite regulation and energy homeostasis [45,46]. Interestingly, in the current study, participants in the lowest E-DII tertile (T1) also showed a significantly higher consumption of fibre along with micronutrients, including vitamin A, beta-carotene, vitamin C, iron, thiamine, riboflavin, niacin, vitamin B6, folic acid, zinc, magnesium, and selenium, as compared to participants in T3, which had a significantly higher intake of total fat, SFA, and MUFA. A Mediterranean diet advocating for fibre sources from many fruits, vegetables, legumes, and grains has shown anti-inflammatory effects when compared with a typical Western diet rich in total fat and SFA but low in fibre [18]. Therefore, this study suggests the potential of advocating an anti-inflammatory diet, such as a Mediterranean or plant-based diet, to improve body fat and subsequently inflammatory status among breast cancer patients.

To the best of our knowledge, this is the first study to report the association between diet inflammatory index and body fat among the breast cancer population in Malaysia. By including only the pre-treatment group, the confounding effect of oncological treatment on weight changes, adiposity gain, and inflammatory status can be removed. However, this study has some limitations. First, the results of the current study were limited owing to the cross-sectional nature of the study. Second, the DII was calculated from the FFQ data, in which the possibility of a recall bias cannot be rejected. Third, this is not a comprehensive E-DII utilizing all 45 food parameters due to current FFQ limitations; nevertheless, the 24 food parameters employed in this study exceeded the minimum to compute E-DII scores because a previous study had calculated the DII score with only 18 food parameters [47]. Therefore, we recommend future studies based on interventional designs to elucidate the association between diet-induced inflammation and obesity, as well as to include measurements of specific inflammatory markers, such as interleukins, to better define the real prognostic impact of a pro-inflammatory diet and excessive body fat among breast cancer patients.

## 5. Conclusion

In this study, a higher E-DII was associated with increased body fat percentage, suggesting the potential of advocating an anti-inflammatory diet to combat obesity among newly diagnosed breast cancer patients, particularly pre-menopausal women. Future studies are recommended to validate this finding and to expound the prognostic impact of a pro-inflammatory diet and excessive body fat on inflammation in breast cancer.

## Data Availability

The data supporting the findings of this study are available from the corresponding author, Zalina Abu Zaid, upon reasonable request.

## Appendix

**Table 1.** Sociodemographic and medical characteristics of study participants according to E-DII tertile.

Characteristics	Tertile of E-DII			Statistical (X^2^/ F)	*p*-value
	**Tertile 1** **(≤−0.26)** **Mean ± SD** **(−0.97 ± 0.834)** ***n* = 41** ***n* (%)**	**Tertile 2** **(−0.27 – 1.36)** **Mean ± SD** **(0.85 ± 0.326)** ***n* = 42** ***n* (%)**	**Tertile 3** **(≥1.37)** **Mean ± SD** **(2.15 ± 0.584)** ***n* = 41** ***n* (%)**		
**Demographic**					
**Age, years (median)** ^a^	49 (42.5, 58.5)	48 (42.0, 61.0)	52 (43.0, 60.0)	0.517	0.772
**Age, years** ^b^					
<50 years old	21 (33.9)	23 (37.1)	18 (29.0)	1.015	0.602
≥50 years old	20 (32.3)	19 (30.6)	23 (37.1)		
					
**Education** ^b^				0.341	0.843
High school or less	27 (33.8)	28 (35.0)	25 (31.2)		
College or university	14 (31.8)	14 (31.8)	16 (36.4)		
					
**Ethnicity** ^b^				3.76	0.709
Malay	28 (32.6)	29 (33.7)	29 (33.7)		
Chinese	10 (45.5)	7 (31.8)	5 (22.7)		
Indian	3 (21.4)	5 (35.7)	6 (42.9)		
Others	0 (0)	1 (50.0)	1 (50.0)		
					
**Marital Status** ^b^				2.067	0.356
Married	29 (29.8)	34 (35.1)	34 (35.1)		
Single/ Widowed/ Divorced	12 (44.5)	8 (29.6)	7 (25.9)		
					
**Household Income, RM (median)** ^a^	3000 (2500, 6000)	4500 (1950, 6625)	4000 (1425, 8000)	0.189	0.91
**Household Income, RM** ^b^				2.961	0.228
<5000	27 (40.9)	19 (28.8)	20 (30.3)		
≥ 5000	12 (26.7)	19 (42.2)	14 (31.1)		
Prefer not to say	2 (15.4)	4 (30.8)	7 (53.8)		
					
**Menopause Status** ^b^				2.237	0.327
Pre-menopausal	23 (34.8)	25 (37.9)	18 (27.3)		
Post-menopausal	18 (31.0)	17 (29.3)	23 (39.7)		
					
**Smoking history** ^b^				3.184	0.203
Never	34 (37.4)	30 (33.0)	27 (29.7)		
Active/ Second-hand smoker	7 (21.2)	12 (36.4)	14 (42.4)		
					
**Comorbidities** ^b^					
NKMI	27 (38.0)	25 (35.2)	19 (26.8)	3.322	0.19
T2DM	3 (18.8)	5 (31.3)	8 (50.0)	2.769	0.25
Hypertension	11 (26.2)	14 (33.3)	17 (40.5)	1.968	0.374
Dyslipidemia	10 (38.5)	7 (26.9)	9 (34.6)	0.783	0.676
					
**Staging** ^b^				1.074	0.585
1 to 2	20 (37.0)	17 (31.5)	17 (31.5)		
3 to 4	17 (28.3)	23 (38.3)	20 (33.3)		
Yet to rule out	4 (40.0)	2 (20.0)	4 (40.0)		
					

^a^ Kruskal-Wallis test;.

^b^ Chi Square test for proportions.

**Table 2.** Body composition and biochemical parameters, physical activity and functional status of study participants according to E-DII tertiles.

Characteristics	Tertile of E-DII			Statistical (J-T)	*p*-value for trend
	**T1** **(≤ −0.26)** **Mean ± SD** **(−0.97 ± 0.834)** ***n* = 41** ***n* (%)/** **Mean ± SD/** **Median (IQR)**	**T2** **(−0.27 −1.36)** **Mean ± SD** **(0.85 ± 0.326)** ***n* = 42** ***n* (%)/** **Mean ± SD/** **Median (IQR)**	**T3** **(≥1.37)** **Mean ± SD** **(2.15 ± 0.584)** ***n* = 41** ***n* (%)/** **Mean ± SD/** **Median (IQR)**		
**Body Composition**					
BMI (kg/m2)^a^	25.5 ± 4.61	26.0 ± 4.54	27.8 ± 5.37	1.902	0.057
Body weight (kg)^a^	61.0 ± 11.26	64.3 ± 12.54	67.6 ± 12.52	2.306	0.021*
Body Fat Percentage^a^	36.0 ± 6.69	36.8 ± 6.43	39.1 ± 6.87	2.021	0.043*
FM/FFM^a^	0.58 ± 0.165	0.60 ± 0.167	0.66 ± 0.191	2.007	0.045*
Muscle Mass^a^	36.1 ± 3.31	37.6 ± 4.18	38.0 ± 3.52	2.338	0.019*
Visceral Fat^a^	7.0 (5.0, 9.5)	8.0 (5.8, 9.0)	9.0 (7.0, 10.5)	2.185	0.029*
					
**Biochemical**					
Albumin^a^	40.0 (42.0, 44.0)	41.0 (39.0, 43.3)	41.0 (40.0, 43.0)	−1.19	0.234
Haemoglobin^a^	12.70 (11.95, 13.55)	12.55 (11.90, 13.25)	12.50 (11.73, 13.00)	−1.132	0.258
White blood cell^a^	6.7 ± 1.68	7.2 ± 2.00	7.5 ± 1.78	2.281	0.023*
Neutrophil^a^	3.65 (2.87, 4.82)	4.00 (3.15, 5.25)	3.92 (3.17, 5.11)	0.771	0.441
Lymphocyte^a^	2.1 ± 0.62	2.2 ± 0.69	2.5 ± 0.71	2.68	0.007**
					
**Handgrip strength, kg (Median)** ^a^	12.7 (10.6, 15.6)	13.1 (10.5, 16.7)	11.1 (8.4, 13.8)	−1.544	0.123
					
**Physical Activity**, **METs/week**^b^					
Sedentary	15 (42.9%)	12 (34.3%)	8 (22.9%)	2.953	0.228
Moderate-High	26 (29.2%)	30 (33.7%)	33 (37.1%)		

^a^ Jonckheere–Terpstra test; ^b^Chi Square test for proportions.

* *p* < 0.05, ***p* < 0.01.

**Table 3.** Intakes of 24 food parameters according to E-DII tertiles.

Food parameter	Tertile of E-DII			Statistical (F/ H)	p-value
	**T1** **(≤−0.26)** **Mean ± SD** **(−0.97 ± 0.834)** ***n* = 41** ***n* (%)/** **Mean ± SD/** **Median (IQR)**	**T2** **(−0.27 −1.36)** **Mean ± SD** **(0.85 ± 0.326)** ***n* = 42** ***n* (%)/** **Mean ± SD/** **Median (IQR)**	**T3** **(≥1.37)** **Mean ± SD** **(2.15 ± 0.584)** ***n* = 41** ***n* (%)/** **Mean ± SD/** **Median (IQR)**		
**DII** ^a^	−0.29 (-1.12, 0.64)	1.24 (0.71, 1.60)	2.13 (1.62, 2.63)	73.054	<0.001***
Energy^b^	1902 ± 456.1	1871 ± 554.1	1850 ± 443.2	0.117	0.89
Protein^b^	81.6 ± 22.09	71.5 ± 26.21	70.8 ± 23.87	2.586	0.08
Carbohydrate^b^	261.6 ± 68.86	255.8 ± 76.13	231.1 ± 62.45	2.238	0.111
Total Fat^a^	57.5 (46.2, 68.9)	59.0 (47.3, 79.1)	69.8 (56.7, 83.1)	7.447	0.024*
SFA^b^	21.0 ± 6.63	24.0 ± 9.45	27.5 ± 7.03	6.932	0.001*
MUFA^b^	24.0 ± 8.70	24.9 ± 8.44	28.4 ± 7.07	3.325	0.039*
PUFA^a^	11.9 (9.2, 14.6)	11.8 (9.4, 15.8)	11.7 (9.8, 14.9)	0.024	0.988
Cholesterol^a^	292.5 (206.3, 455.6)	251.5 (188.8, 350.4)	302.1 (190.6, 416.3)	1.72	0.423
Vitamin A^a^	876.71 (616.42, 1119.63)	610.89 (496.35, 786.04)	432.27 (362.17, 588.80)	33.416	<0.001***
B-carotene^a^	3328.35 (2515.61, 4639.54)	2037.39 (1399.82, 2884.69)	1511.64 (1143.44, 1713.12)	47.621	<0.001***
Vitamin C^a^	271.00 (213.31, 350.34)	157.02 (109.17, 188.38)	98.80 (85.37, 151.88)	53.689	<0.001***
Iron^a^	16.49 (13.08, 21.01)	15.24 (10.91, 18.64)	13.40 (10.48, 17.41)	8.972	0.011*
Thiamine^b^	1.49 ± 0.532	1.23 ± 0.485	1.01 ± 0.409	10.199	<0.001***
Riboflavin^b^	2.01 ± 0.701	1.67 ± 0.702	1.47 ± 0.643	6.574	0.002**
Niacin^b^	15.26 ± 5.258	12.79 ± 5.581	11.85 ± 4.127	5.014	0.008**
Vitamin B6^a^	2.15 (1.74, 2.70)	1.64 (1.29, 2.31)	1.68 (1.21, 2.06)	15.597	<0.001***
Folic acid^a^	298.29 (223.91, 402.67)	241.62 (179.61, 310.41)	176.23 (136.97, 260.40)	16.433	<0.001***
Vitamin B12^a^	5.35 (3.08, 7.88)	3.78 (2.30, 6.71)	4.06 (2.56, 5.31)	4.801	0.091
Zinc^a^	10.59 (8.15, 15.30)	8.56 (6.91, 12.02)	8.38 (6.05, 9.69)	13.533	0.001**
Magnesium^b^	324.42 ± 84.678	257.18 ± 86.000	224.93 ± 71.555	16.093	<0.001***
Selenium^a^	126.74 (92.80, 181.04)	104.63 (84.09, 147.76)	110.48 (74.90, 138.58)	7.354	0.025*
Fiber^a^	13.9 (8.2, 19.8)	9.0 (6.1, 12.9)	6.0 (4.9, 8.6)	31.512	<0.001***
Caffeine^a^	27.26 (0.51, 80.35)	80.35 (20.45, 140.74)	47.73 (11.84, 114.76)	7.899	0.019*
Alcohol^a^	0 (0, 0)	0 (0, 0)	0 (0, 0)	4.695	0.096

^a^ Kruskal-Wallis test; ^b^One way ANOVA.

* *p* < 0.05; ***p* < 0.01; ****p* < 0.001.

**Table 4.** The ORs and 95% CIs for the associations between the E-DII and body fat (*N* = 124).

	E-DII score [ref: Tertile 1 (≤−0.26)]							
	Tertile 2 (−0.27 −1.36)				Tertile 3 (≥1.37)			
	Adjusted Coefficient	**OR** **(95% CI)**	SE	*p*-value	Adjusted Coefficient	**OR** **(95% CI)**	SE	*p*-value
**Body Fat Percentage**								
**Model 1**	0.644	1.905 (0.785, 4.624)	0.453	0.154	1.083	2.952 (1.154, 7.556)	0.479	0.024*
**Model 2**	0.680	1.974 (0.786, 4.954)	0.470	0.148	1.194	3.300 (1.223, 8.901)	0.506	0.018*
**Model 3**	0.792	2.208 (0.828, 5.885)	0.500	0.113	1.385	3.996 (1.311, 12.174)	0.568	0.015*

Model 1: energy-adjusted DII.

Model 2: adjusted for energy intake and cancer stage.

Model 3: further adjustments for age, physical activity (continuous), ethnicity, smoking history, comorbidities of NKMI, T2DM, hypertension, and dyslipidemia.

OR, Odds ratio; SE Standard error; CI Confidence interval; E-DII, energy-adjusted dietary inflammatory index.

* *p* < 0.05.

**Table 5.** The ORs and 95% CIs for the associations between the E-DII and body fat according to menopausal status (*N* = 124).

	E-DII score [ref: Tertile 1 (≤−0.26)]							
	Pre-menopausal (*n* = 66)				Post-menopausal (*n* = 58)			
	Tertile 3 (≥1.37)				Tertile 3 (≥1.37)			
	Adjusted Coefficient	**OR** **(95% CI)**	SE	*p*-value	Adjusted Coefficient	**OR** **(95% CI)**	SE	*p*-value
**Body Fat Percentage**								
**Model 1**	1.522	4.583 (1.038, 20.240)	0.758	0.045*	0.827	2.286 (0.634, 8.234)	0.654	0.206
**Model 2**	1.614	5.022 (1.053, 23.948)	0.797	0.043*	1.080	2.945 (0.741, 11.699)	0.704	0.125
**Model 3**	1.977	7.218 (1.148, 45.366)	0.938	0.035*	1.043	2.838 (0.494, 16.311)	0.892	0.242

Model 1: energy-adjusted DII.

Model 2: adjusted for energy, cancer stage.

Model 3: further adjustments for age, physical activity (continuous), ethnicity, smoking history, comorbidities of NKMI, T2DM, hypertension, and dyslipidemia.

OR, Odds ratio; SE Standard error; CI Confidence interval; E-DII, energy-adjusted dietary inflammatory index.

* *p* < 0.05.

## References

[1] Sung H, Ferlay J, Siegel RL, et al. Global cancer statistics 2020: GLOBOCAN estimates of incidence and mortality worldwide for 36 cancers in 185 countries. CA Cancer J Clin. 2021;71(3):1–11. doi: 10.3322/caac.21660. 33538338

[2] Lobstein T, Brinsden H, Neveux M. 2022). World Obesity Atlas 2022, *World Obesity Federation*. United Kingdom. Retrieved from https://policycommons.net/artifacts/2266990/world_obesity_atlas_2022_web/3026660/?utm_medium=email&utm_source=transaction on 27 Jul 2023. CID: 20.500.12592/74dsjp.

[3] Abe R, Kumagai N, Kimura M, et al. Biological characteristics of breast cancer in obesity. Tohoku J Exp Med. 1976;120(4):351–359. doi: 10.1620/tjem.120.351. 1014000

[4] Pati S, Irfan W, Jameel A, et al. Obesity and cancer: a current overview of epidemiology, pathogenesis, outcomes, and management. Cancers (Basel). 2023;15(2):485. doi: 10.3390/cancers15020485. 36672434 PMC9857053

[5] Jiralerspong S, Goodwin PJ. Obesity and breast cancer prognosis: evidence, challenges, and opportunities. J Clin Oncol. 2016;34(35):4203–4216. doi: 10.1200/JCO.2016.68.4480. 27903149

[6] Deshmukh SK, Srivastava SK, Poosarla T, et al. Inflammation, immunosuppressive microenvironment and breast cancer: opportunities for cancer prevention and therapy. Ann Transl Med. 2019;7(20):593–593. doi: 10.21037/atm.2019.09.68. 31807574 PMC6861749

[7] Crespi E, Bottai G, Santarpia L. Role of inflammation in obesity-related breast cancer. Curr Opin Pharmacol. 2016;31:114–122. doi: 10.1016/j.coph.2016.11.004. 27889687

[8] Lee S, Quiambao AL, Lee J, et al. Dietary inflammatory index and risk of breast cancer based on hormone receptor status: a case-control study in korea. Nutrients. 2019;11(8):1949. doi: 10.3390/nu11081949. 31430979 PMC6723443

[9] Meyer D, Pastor-Villaescusa B, Michel S, et al. Associations between circulating obesity-related biomarkers and prognosis in female breast cancer survivors: a systematic review of observational data in women enrolled in lifestyle intervention trials. BMC Cancer. 2022;22(1):1187. doi: 10.1186/s12885-022-10274-3. 36401194 PMC9673384

[10] Iyengar NM, Chen IC, Zhou XK, et al. Adiposity, inflammation, and breast cancer pathogenesis in asian women. Cancer Prev Res (Phila). 2018;11(4):227–236. doi: 10.1158/1940-6207.CAPR-17-0283. 29222346 PMC5882588

[11] Mullooly M, Yang HP, Falk RT, et al. Relationship between crown-like structures and sex-steroid hormones in breast adipose tissue and serum among postmenopausal breast cancer patients. Breast Cancer Res. 2017;19(1):8. doi: 10.1186/s13058-016-0791-4. 28103902 PMC5244534

[12] Irwin ML, McTiernan A, Baumgartner RN, et al. Changes in body fat and weight after a breast cancer diagnosis: influence of demographic, prognostic and lifestyle factors. J Clin Oncol. 2005;23(4):774–782. doi: 10.1200/JCO.2005.04.036. 15681521 PMC3000612

[13] Xu S, Xue Y. Pediatric obesity: causes, symptoms, prevention and treatment. Exp Ther Med. 2016;11(1):15–20. doi: 10.3892/etm.2015.2853. 26834850 PMC4726862

[14] Garaulet M, Ordovás JM, Madrid JA. The chronobiology, etiology and pathophysiology of obesity. Int J Obes (Lond). 2010;34(12):1667–1683. doi: 10.1038/ijo.2010.118. 20567242 PMC4428912

[15] Duvigneaud N, Wijndaele K, Matton L, et al. Dietary factors associated with obesity indicators and level of sports participation in flemish adults: a cross-sectional study. Nutr J. 2007;6(1):26. doi: 10.1186/1475-2891-6-26. 17883880 PMC2094711

[16] Vahid F, Bourbour F, Gholamalizadeh M, et al. A pro-inflammatory diet increases the likelihood of obesity and overweight in adolescent boys: a case–control study. Diabetol Metab Syndr. 2020;12(1):29. doi: 10.1186/s13098-020-00536-0. 32292493 PMC7140561

[17] Shivappa N, Steck SE, Hurley TG, et al. Designing and developing a literature-derived, population-based dietary inflammatory index. Public Health Nutr. 2014;17(8):1689–1696. doi: 10.1017/S1368980013002115. 23941862 PMC3925198

[18] Cavicchia PP, Steck SE, Hurley TG, et al. A new dietary inflammatory index predicts interval changes in serum high-sensitivity C-reactive protein. J Nutr. 2009;139(12):2365–2372. doi: 10.3945/jn.109.114025. 19864399 PMC2777480

[19] Saghafi-Asl M, Mirmajidi S, Asghari Jafarabadi M, et al. The association of dietary patterns with dietary inflammatory index, systemic inflammation, and insulin resistance, in apparently healthy individuals with obesity. Sci Rep. 2021;11(1):7515. doi: 10.1038/s41598-021-86993-7. 33824355 PMC8024341

[20] Ruiz-Canela M, Zazpe I, Shivappa N, et al. Dietary inflammatory index and anthropometric measures of obesity in a population sample at high cardiovascular risk from the PREDIMED (PREvención con DIeta MEDiterránea) trial. Br J Nutr. 2015;113(6):984–995. doi: 10.1017/S0007114514004401. 25720588 PMC4870040

[21] Moreno-Aliaga MJ, Campión J, Milagro FI, et al. Adiposity and proinflammatory state: the chicken or the egg. Adipocytes. 2005;1:1–16.

[22] Lwanga SK, Lemeshow S, World Health Organization. 1991. Sample size determination in health studies: a practical manual. Geneva: World Health Organization.

[23] Song D, Kim J, Kang M, et al. Association between the dietary inflammatory index and bone markers in postmenopausal women. PLoS One. 2022;17(3):e0265630. doi: 10.1371/journal.pone.0265630. 35298570 PMC8929634

[24] World Health Organization. 2000. Obesity: preventing and managing the global epidemic: report of a WHO consultation. Geneva: World Health Organization. 11234459

[25] World Health Organization. 1995. Physical status: the use of and interpretation of anthropometry, report of a WHO expert committee. Geneva: World Health Organization. 8594834

[26] Ministry of Health Malaysia. 2014. National health and morbidity survey 2014: malaysian adult nutrition survey (MANS). Volume I: methodology and general findings. Putrajaya (Malaysia): Ministry of Health Malaysia.

[27] Abdul Manaf Z, Shahar S, Safii NS, et al. 2015). Atlas of food exchanges & portion sizes. *3rd ed.* Kuala Lumpur, Malaysia. MDC.

[28] Hébert JR, Shivappa N, Wirth MD, et al. Perspective: the dietary inflammatory index (DII)—lessons learned, improvements made, and future directions. Adv Nutr. 2019;10(2):185–195. doi: 10.1093/advances/nmy071. 30615051 PMC6416047

[29] Harmon BE, Wirth MD, Boushey CJ, et al. The dietary inflammatory index is associated with colorectal cancer risk in the multiethnic cohort. J Nutr. 2017;147(3):430–438. doi: 10.3945/jn.116.242529. 28179489 PMC5320401

[30] Craig CL, Marshall AL, Sjöström M, et al. International physical activity questionnaire: 12-country reliability and validity. Med Sci Sports Exerc. 2003;35(8):1381–1395. doi: 10.1249/01.MSS.0000078924.61453.FB. 12900694

[31] Garvey WT, Mechanick JI. Proposal for a scientifically correct and medically actionable disease classification system (ICD) for obesity. Obesity (Silver Spring). 2020;28(3):484–492. doi: 10.1002/oby.22727. 32090513 PMC7045990

[32] Romero-Corral A, Somers VK, Sierra-Johnson J, et al. Accuracy of body mass index to diagnose obesity in the US adult population. Int J Obes (Lond). 2008;32(6):959–966. doi: 10.1038/ijo.2008.11. 18283284 PMC2877506

[33] Bergqvist M, Elebro K, Sandsveden M, et al. Effects of tumor-specific CAP1 expression and body constitution on clinical outcomes in patients with early breast cancer. Breast Cancer Res. 2020;22(1):67. doi: 10.1186/s13058-020-01307-5. 32560703 PMC7304201

[34] Pang Y, Wei Y, Kartsonaki C. Associations of adiposity and weight change with recurrence and survival in breast cancer patients: a systematic review and meta-analysis. Breast Cancer. 2022;29(4):575–588. doi: 10.1007/s12282-022-01355-z. 35579841 PMC9226105

[35] Dee A, McKean-Cowdin R, Neuhouser ML, et al. DEXA measures of body fat percentage and acute phase proteins among breast cancer survivors: a cross-sectional analysis. BMC Cancer. 2012;12(1):343. doi: 10.1186/1471-2407-12-343. 22873489 PMC3500231

[36] Kenđel Jovanović G, Mrakovcic-Sutic I, Pavičić Žeželj S, et al. The efficacy of an energy-restricted anti-inflammatory diet for the management of obesity in younger adults. Nutrients. 2020;12(11):3583. doi: 10.3390/nu12113583. 33266499 PMC7700374

[37] Ramallal R, Toledo E, Martínez JA, et al. Inflammatory potential of diet, weight gain, and incidence of overweight/obesity: the SUN cohort. Obesity (Silver Spring). 2017;25(6):997–1005. doi: 10.1002/oby.21833. 28544794

[38] Mehrdad M, Vahid F, Shivappa N, et al. High dietary inflammatory index (DII) scores increase odds of overweight in adults with rs9939609 polymorphism of FTO gene. Clin Nutr ESPEN. 2021;42:221–226. doi: 10.1016/j.clnesp.2021.01.034. 33745583

[39] Shivappa N, Blair CK, Prizment AE, et al. Prospective study of the dietary inflammatory index and risk of breast cancer in postmenopausal women. Mol Nutr Food Res. 2017;61(5):1600592. doi: 10.1002/mnfr.201600592. PMC5415414 27860246

[40] Park S, Na W, Sohn C. Relationship between osteosarcopenic obesity and dietary inflammatory index in postmenopausal korean women: 2009 to 2011 korea national health and nutrition examination surveys. J Clin Biochem Nutr. 2018;63(3):211–216. doi: 10.3164/jcbn.18-10. 30487671 PMC6252300

[41] Gholamalizadeh M, Ahmadzadeh M, BourBour F, et al. Associations between the dietary inflammatory index with obesity and body fat in male adolescents. BMC Endocr Disord. 2022;22(1):115. doi: 10.1186/s12902-022-01001-x. 35501761 PMC9059349

[42] Barragán-Vázquez S, Ariza AC, Ramírez Silva I, et al. Pro-Inflammatory diet is associated with adiposity during childhood and with adipokines and inflammatory markers at 11 years in mexican children. Nutrients. 2020;12(12):3658. doi: 10.3390/nu12123658. 33261143 PMC7760203

[43] Bagheri S, Zolghadri S, Stanek A. Beneficial effects of anti-inflammatory diet in modulating gut microbiota and controlling obesity. Nutrients. 2022;14(19):3985. doi: 10.3390/nu14193985. 36235638 PMC9572805

[44] Tumminia A, Vinciguerra F, Parisi M, et al. Adipose tissue, obesity and adiponectin: role in endocrine cancer risk. Int J Mol Sci. 2019;20(12):2863. doi: 10.3390/ijms20122863. 31212761 PMC6628240

[45] Byrne CS, Chambers ES, Morrison DJ, et al. The role of short chain fatty acids in appetite regulation and energy homeostasis. Int J Obes (Lond). 2015;39(9):1331–1338. doi: 10.1038/ijo.2015.84. 25971927 PMC4564526

[46] Topping DL, Clifton PM. Short-chain fatty acids and human colonic function: roles of resistant starch and nonstarch polysaccharides. Physiol Rev. 2001;81(3):1031–1064. doi: 10.1152/physrev.2001.81.3.1031. 11427691

[47] Shivappa N, Hébert JR, Steck SE, et al. Dietary inflammatory index and odds of colorectal cancer in a case-control study from Jordan. Appl Physiol Nutr Metab. 2017;42(7):744–749. doi: 10.1139/apnm-2017-0035. 28226219

